# Supplementary material for: Role of Extracellular High-Mobility Group Box-1 as a Therapeutic Target of Gastric Cancer
Source: Int J Mol Sci. 2022 Mar 17;23(6):3264. doi: 10.3390/ijms23063264 (PMC8953630; doi:10.3390/ijms23063264)
Supplement: Supplementary file 1 [file ijms-23-03264-s001.zip › ijms-1572182-supplementary.pdf]

Supplementary Figure S1

(a)

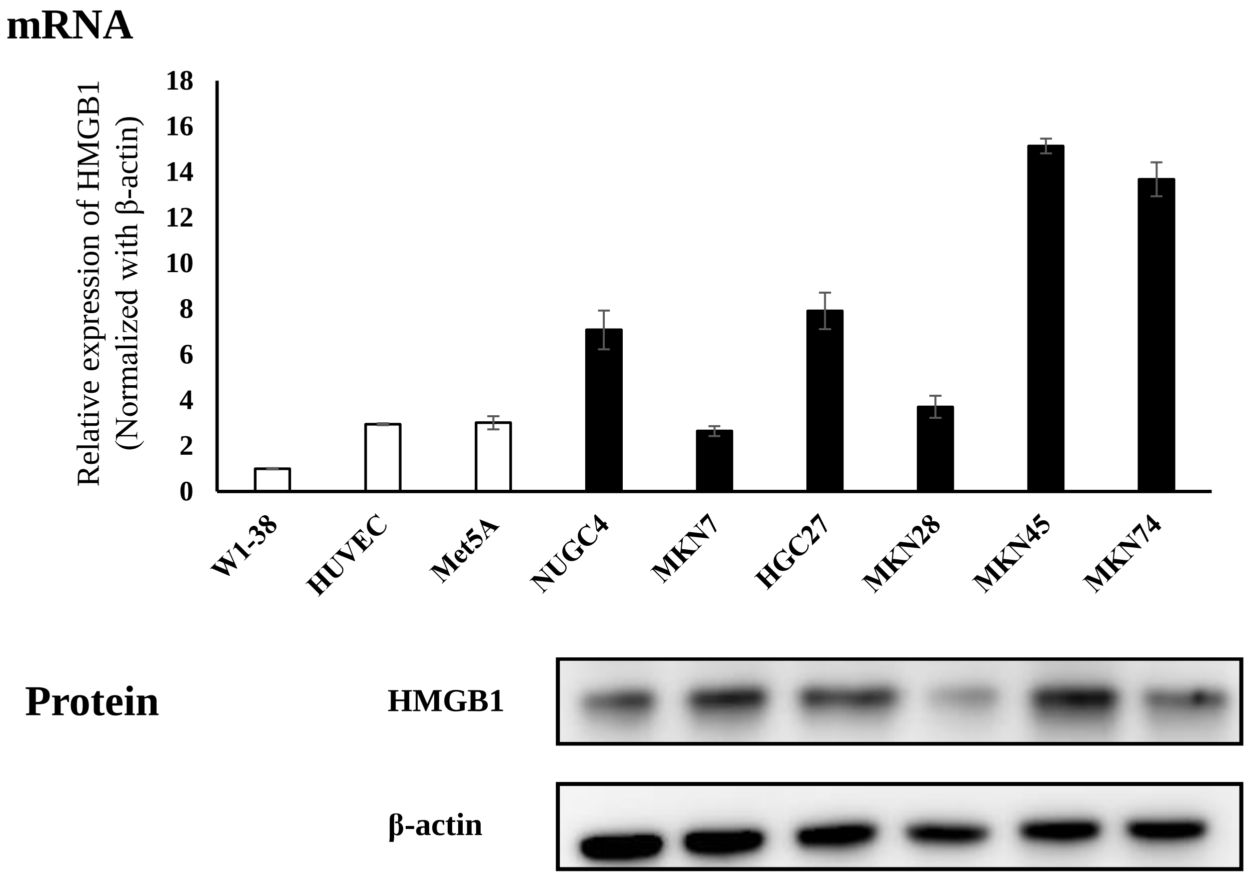

(b)

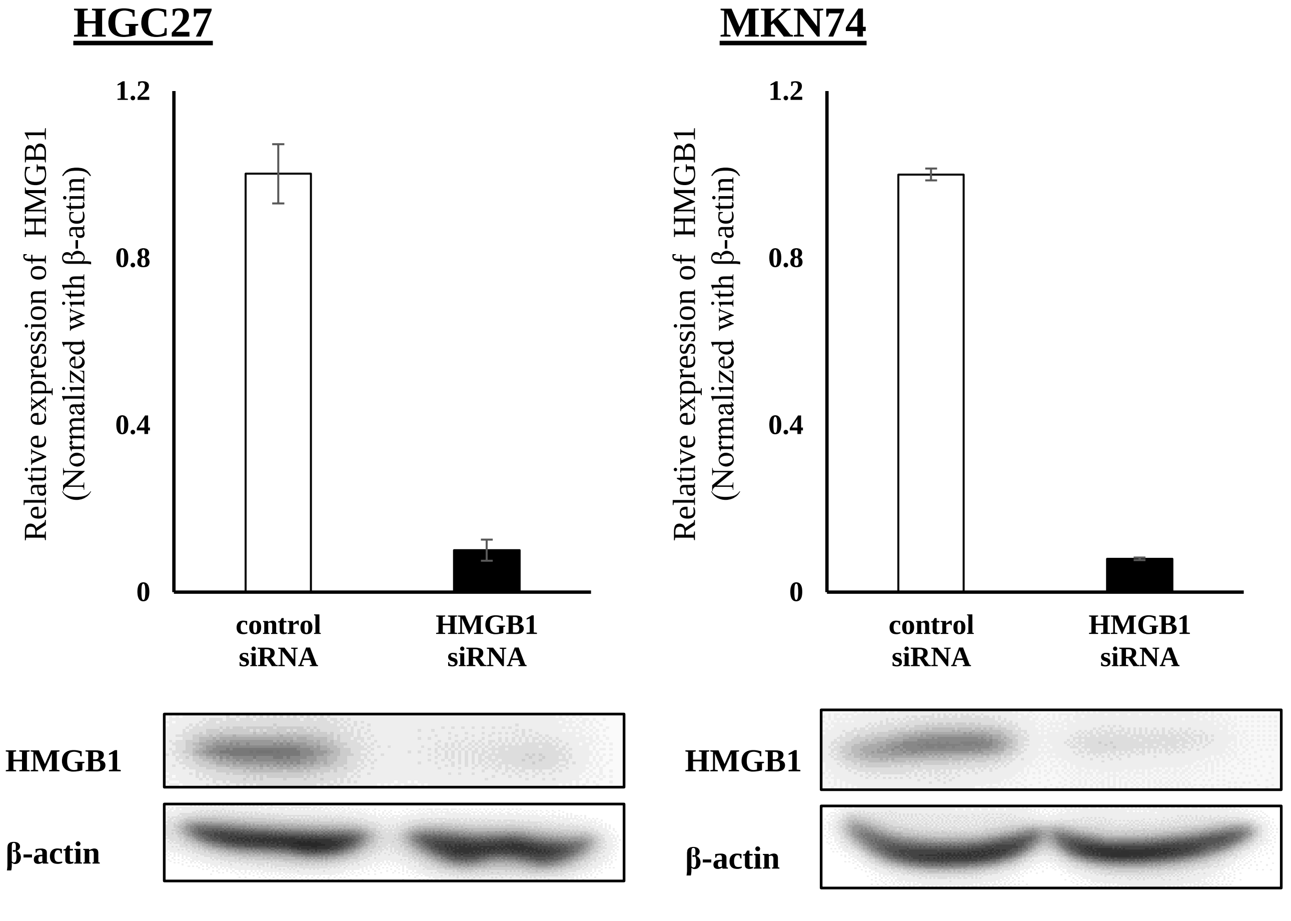

Supplementary Figure S2

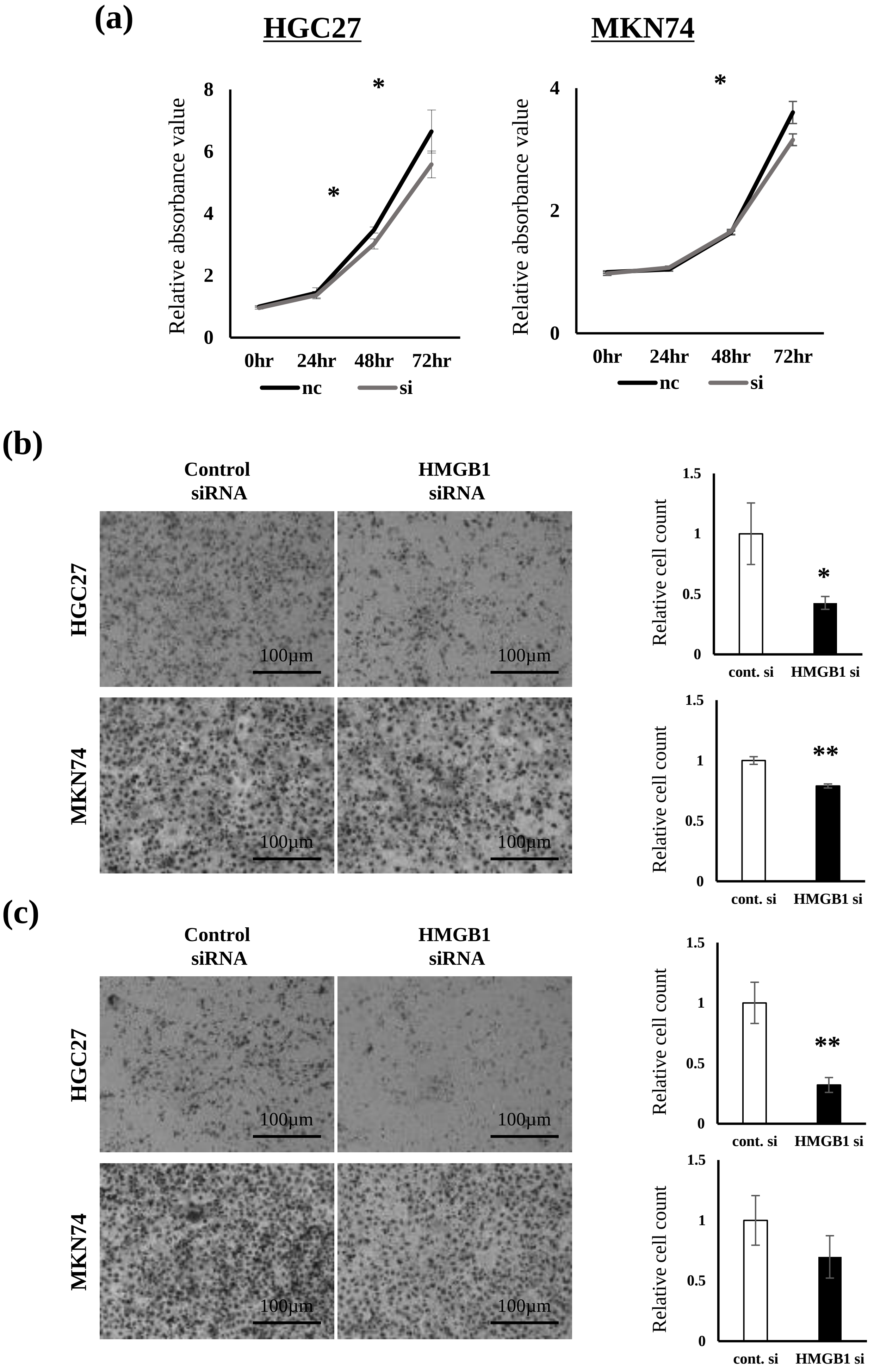

Supplementary Figure S3

(a)

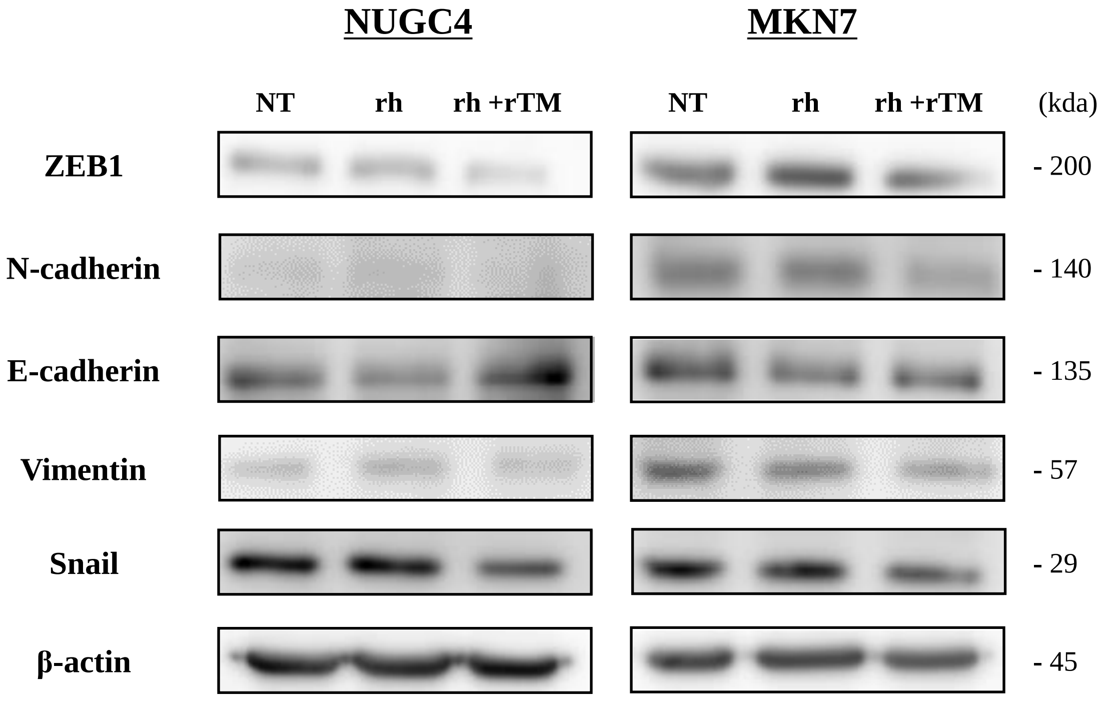

(b)

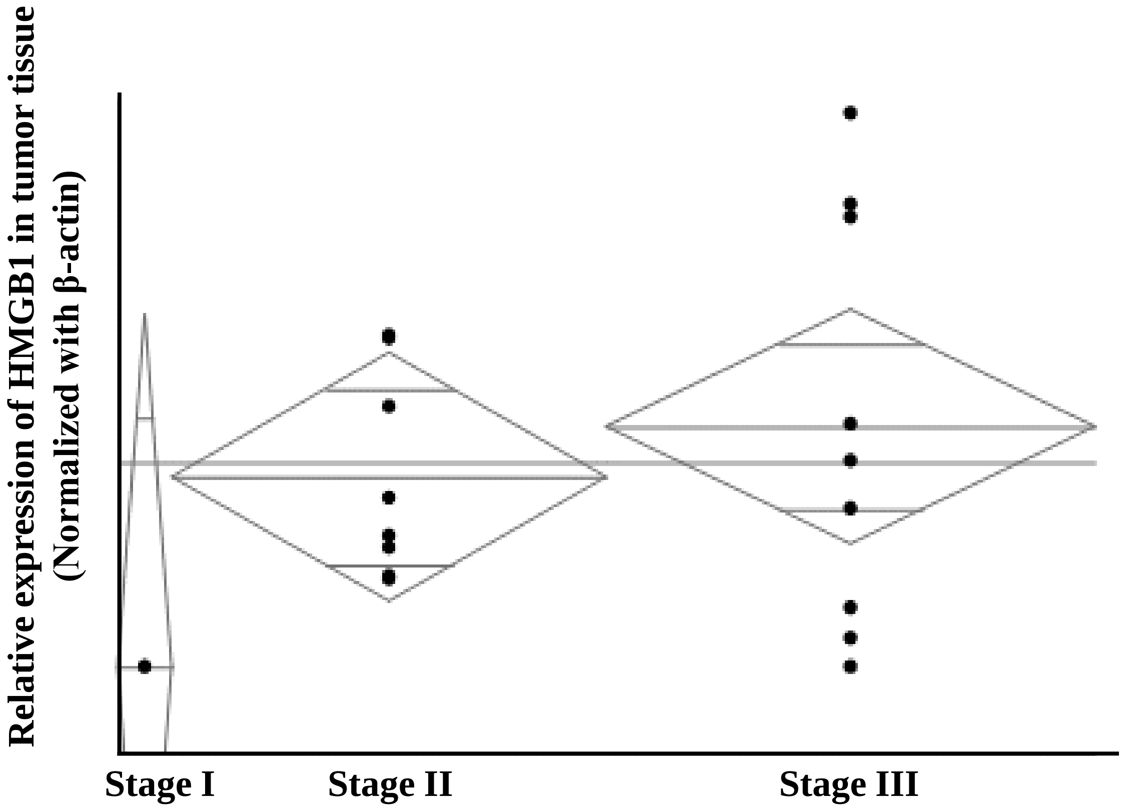

Supplementary Figure S4

(a)

Treatment schedule of the subcutaneous tumor model

Subcutaneous injection of tumor cells
: 0.5×10^6 of NUGC4 cells (n=5, respectively)

i) +PBS only
ii) +rhHMGB1 (0.5 µg/mouse)
iii) +rhHMGB1 (0.5 µg/mouse)+ rTM (0.25 µg/mouse)

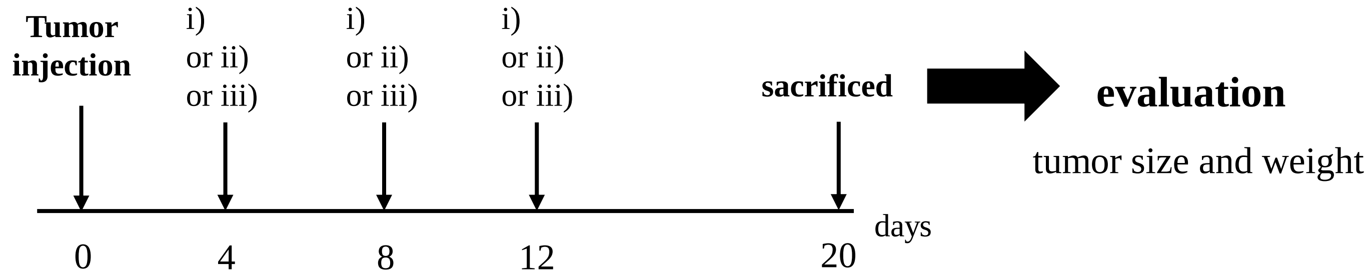

(b)

Treatment schedule of the liver metastasis model

Tumor cell injection into the spleen via the splenic vein
: 0.5×10^6 of NUGC4 cells (n=5, respectively)

i) +PBS only
ii) +rhHMGB1 (0.5 µg/mouse)
iii) +rhHMGB1 (0.5 µg/mouse)+ rTM (0.25 µg/mouse)

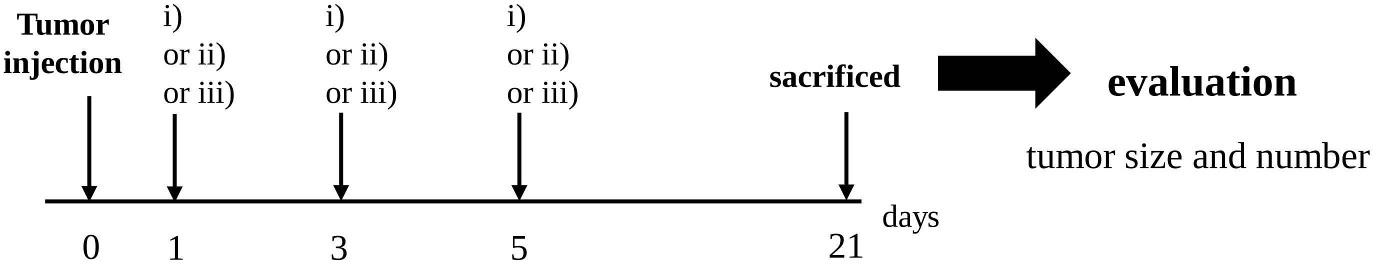

Supplementary Figure S5

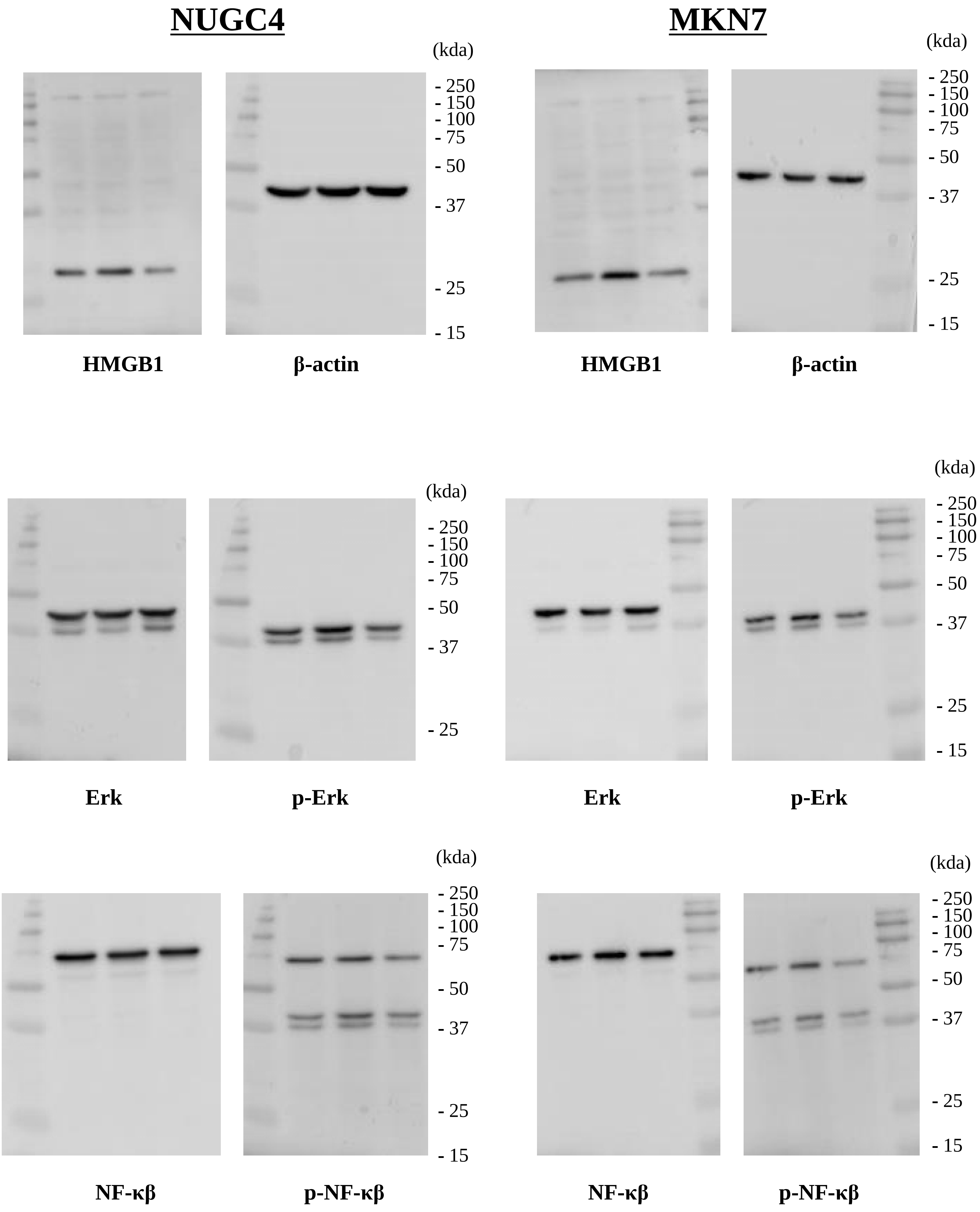

Supplementary Figure S6

(a)

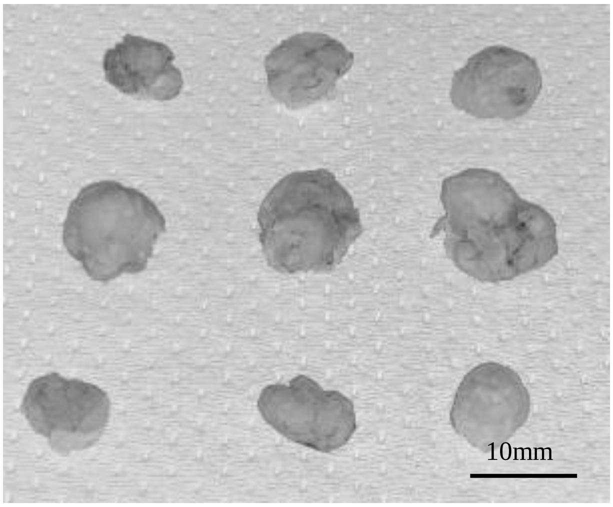

	volume (mm^3)			average	weight (μg)			average
NT	401	478	429	436	465	524	470	486
rhHMGB1	758	850	982	863	847	985	904	912
rhHMGB1+rTM	587	625	736	649	609	651	726	662

(b)

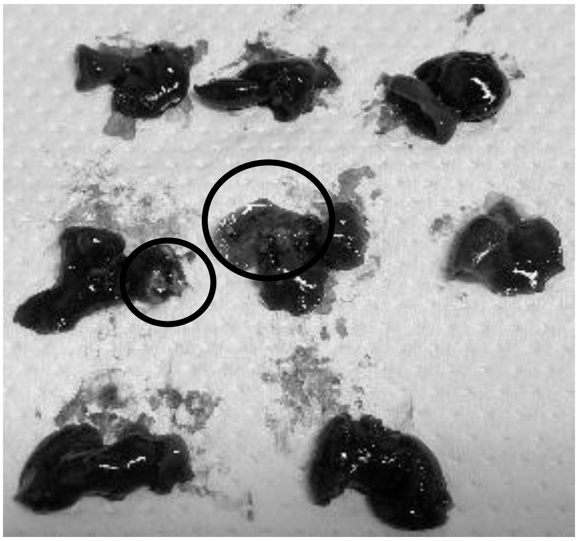

**Treatment**	**Number of mice with metastasis**
NT	0/3
+rhHMGB1	2/3
+rhHMGB1 +rTM	0/2

Table S1 Clinicopathological characteristics of patients included in the present study.

**Variables**		**Number (%)**
age (years)	mean ± SD	68.3 ± 11.4
sex	Male	38 (67.8)
	Female	18 (32.2)
BMI (kg/m^2)	mean ± SD	22.0 ± 3.6
Location	U	10 (17.8)
	M	22 (39.3)
	L	24 (42.9)
Type	0-2	30 (53.6)
	3-4	26 (46.4)
size (mm)	mean ± SD	57.2 ± 34.7
Histology	Well	28 (50.0)
	Poor	28 (50.0)
pT[a] factor	1	9 (16.1)
	2	12 (21.4)
	3	20 (35.7)
	4	15 (26.8)
pN[a] factor	0	17 (30.4)
	1	15 (26.8)
	2	13 (23.2)
	3	11 (19.6)
pStage[a]	1	13 (23.2)
	2	20 (35.7)
	3	23 (41.1)
ly[a]	0	15 (26.7)
	1-3	41 (73.2)
v[a]	0	17 (30.3)
	1-3	39 (69.7)

[a] According to the 8th edition of the UICC/Staging System for Tumors, Nodules, and Metastases

[b] p-values are from the Log-rank test

BMI; body mass index, U; upper, M; middle, L; lower
